# Microbiability and microbiome-wide association analyses of feed efficiency and performance traits in pigs

**DOI:** 10.1186/s12711-022-00717-7

**Published:** 2022-04-25

**Authors:** Amir Aliakbari, Olivier Zemb, Laurent Cauquil, Céline Barilly, Yvon Billon, Hélène Gilbert

**Affiliations:** 1 GenPhySE, Université de Toulouse, INRAE, ENVT, 31320 Castanet-Tolosan, France; 2 GenESI, INRAE, 17700 Surgères, France

## Abstract

**Background:**

The objective of the present study was to investigate how variation in the faecal microbial composition is associated with variation in average daily gain (ADG), backfat thickness (BFT), daily feed intake (DFI), feed conversion ratio (FCR), and residual feed intake (RFI), using data from two experimental pig lines that were divergent for feed efficiency. Estimates of microbiability were obtained by a Bayesian approach using animal mixed models. Microbiome-wide association analyses (MWAS) were conducted by single-operational taxonomic units (OTU) regression and by back-solving solutions of best linear unbiased prediction using a microbiome covariance matrix. In addition, accuracy of microbiome predictions of phenotypes using the microbiome covariance matrix was evaluated.

**Results:**

Estimates of heritability ranged from 0.31 ± 0.13 for FCR to 0.51 ± 0.10 for BFT. Estimates of microbiability were lower than those of heritability for all traits and were 0.11 ± 0.09 for RFI, 0.20 ± 0.11 for FCR, 0.04 ± 0.03 for DFI, 0.03 ± 0.03 for ADG, and 0.02 ± 0.03 for BFT. Bivariate analyses showed a high microbial correlation of 0.70 ± 0.34 between RFI and FCR. The two approaches used for MWAS showed similar results. Overall, eight OTU with significant or suggestive effects on the five traits were identified. They belonged to the genera and families that are mainly involved in producing short-chain fatty acids and digestive enzymes. Prediction accuracy of phenotypes using a full model including the genetic and microbiota components ranged from 0.60 ± 0.19 to 0.78 ± 0.05. Similar accuracies of predictions of the microbial component were observed using models that did or did not include an additive animal effect, suggesting no interaction with the genetic effect.

**Conclusions:**

Our results showed substantial associations of the faecal microbiome with feed efficiency related traits but negligible effects with growth traits. Microbiome data incorporated as a covariance matrix can be used to predict phenotypes of animals that do not (yet) have phenotypic information. Connecting breeding environment between training sets and predicted populations could be necessary to obtain reliable microbiome predictions.

**Supplementary Information:**

The online version contains supplementary material available at 10.1186/s12711-022-00717-7.

## Background

Recent advances in the collection of microbiota information make it possible to analyze the interplay between complex traits and the microbial community of the gastrointestinal tract (GIT) in animals and humans. This is especially essential in the pig industry since previous studies have suggested that the GIT microbiome could contribute to the variability of feed efficiency in pigs [1–3]. From the perspective of quantitative genetics, the effect of the microbiome on a trait can be quantified by estimating the microbiability [4], which is the proportion of phenotypic variance of the trait that is explained by between-animal differences in the microbial community. Estimation of the microbiability requires a microbial relationship matrix between host animals [4]. Using this approach, Camarinha-Silva et al. [3] reported higher estimates of microbiability for feed conversion ratio (FCR) (0.21 ± 0.14) and feed intake (0.16 ± 0.10) than their corresponding heritabilities. Similarly, a recent study revealed that the proportion of variance captured by the microbiome for meat quality and carcass composition traits in crossbred pigs varied over time, with an increased proportion from weaning to off-test for most of the studied traits [5]. This study also reported microbiability (${\mathrm{m}}^{2}$) estimates that were higher than the corresponding heritability (${\mathrm{h}}^{2}$) estimates for some traits, particularly at the off-test stage. In contrast, Tang et al. [6] obtained a lower estimate of ${\mathrm{m}}^{2}$ than that of ${\mathrm{h}}^{2}$ for body weight (BW), average daily gain (ADG), backfat thickness (BFT), and intramuscular fatness, using samples from five different parts of the GIT. Overall, these studies highlight the relevance of variability in the GIT microbiome composition associated with variability in performance traits, which suggests the possibility of predicting future phenotypes based on predicted microbial values ($\widehat{\mathbf{m}}$) [7]. However, in livestock, only a few studies have evaluated the accuracy of phenotype predictions by including microbiota effects in linear mixed models [7, 8]. In addition, similar to genome-wide association studies, microbiome components can be considered as potential markers of the selected complex traits, and their associations can be identified through microbiome-wide association studies (MWAS) [10]. In an early MWAS in the Piétrain pig breed, Camarinha-Silva et al. [3] identified several operational taxonomic units (OTU) with a relative abundance that was significantly associated with ADG, FCR, or feed intake, and they suggested, by analogy to the polygenic determinism of traits, that these traits could have a polymicrobial nature [3]. To the best of our knowledge, there is no additional published literature on this topic in pigs, although there are numerous examples in humans and a few in other livestock species [10, 11]. Given these previous results, we hypothesized that microbiota information can help to predict and manage traits of interest in pigs. The first objective of the present study was to investigate the association of faecal microbial variants with feed efficiency (FCR and residual feed intake (RFI)) and other performance traits, including ADG, BFT, and daily feed intake (DFI). The second objective was to evaluate the accuracy of microbiome predictions of phenotypes of animals that do not (yet) have phenotypic information with the incorporation of a microbiome covariance matrix. We used data from two experimental pig lines that were divergently selected for RFI, which ensured sizeable variability of the traits of interest. In this work, we obtained microbiability estimates for the traits using mixed models with or without accounting for the genetic background of the hosts.

## Methods

### Population structure, studied traits and sampling

Phenotypic records and faecal samples (604 pigs) were collected from the two last generations (G9 and G10) of two experimental French Large White pig lines that were developed over 11 generations of divergent selection for RFI during 18 years at INRAE (UE GenESI, Surgères, France, https://doi.org/10.15454/1.5572415481185847E12), as described in Aliakbari et al. [12]. The structure of the lines and the selection process are described in Aliakbari et al. [13]. After weaning (28 days of age), all pigs were penned in groups of 24, by line and sex. At 10 weeks of age, pigs from each pen were distributed in two growing-finishing pens (n = 12 per pen). There were four pens per room and one or two rooms per contemporary group (CG). Growing-finishing pens were equipped with single-place electronic feeders ACEMA64 (ACEMO, France) to record individual feed intake. A pelleted diet based on cereals and soya bean meal was available ad libitum and contained 10MJ net energy (NE)/kg and 160 g crude protein/kg, with a minimum of 0.80 g digestible Lys/MJ NE. Animals had free access to water at all stages. Complete pedigree information was registered, starting at least one generation before F0 ancestors (founder generation of the divergent lines) until G10.

A set of 157 animals from generation G9 (entire male candidates for selection) had records for feed intake (DFI), feed efficiency traits (FCR and RFI), growth rate (ADG) from 35 to 95 kg of body weight (BW), and live body composition traits at 95 kg BW (BFT). The remaining animals, from G10 (females and castrated males), had records for growth rate, feed efficiency from 10 weeks of age until slaughter (115 kg BW), and backfat thickness at 23 weeks of age (BFT23). Different multiple linear regression equations were used to compute realized RFI for selection candidates versus G10 females and castrated males, considering their test differences, as described in [14]. First, the realized RFI for a selection candidate was defined as the residual for that animal of a multiple regression across selection candidates from G0 to G9 of DFI on ADG and BFT (measured by ultrasound), including the fixed effects of pen size and CG. Then, for all females and castrated males from G1 to G10, the realized RFI was the residual of multiple regression of DFI on average metabolic body weight, ADG, and indicators of body composition (carcass BFT and lean meat content (computed from cut weights) until G9, BFT23 for G10 animals), including the fixed effects of pen size, CG and sex and covariate of BW at the beginning of the test. The FCR was computed based on the corresponding test period of the two groups of animals. Standardized phenotypes of RFI, FCR, DFI, ADG, and BFT were used, as previously proposed in Aliakbari et al. [13]. The descriptive information for these five traits and the sampled G9 and G10 animals is in Table 1.

**Table 1:** Descriptive statistics of the standardized phenotypes for the evaluated production traits

Trait	Number	Min	Max	Average	SD
RFI	522	− 0.38	0.39	0.00	0.15
FCR	548	1.60	3.93	2.78	0.33
DFI	542	1.37	2.95	2.20	0.29
ADG	575	0.51	1.01	0.78	0.08
BFT	541	9.8	46.6	23.3	10.0

*RFI* residual feed intake (kg/day), *FCR* feed conversion ratio (kg/kg), *DFI* daily feed intake (kg/day), *ADG* average daily gain (kg/day), *BFT* backfat thickness (mm)

The faecal samples were collected at 15 weeks of age to obtain the gut microbial information. Immediately after collection, the samples were homogenized and placed in dry ice, before storage at − 80 °C until DNA extraction (see next section).

### Microbial information

The Quick-DNA™ Faecal Microbe Miniprep Kit™ (Zymo Research, Freiburg, Germany) was used to extract microbial DNA based on a 15-min bead-beating step at 30 Hz. PCR amplification of the V3-V4 region of the 16S rRNA gene obtained from diluted genomic DNA was carried out by using two primers: F343 (CTTTCCCTACACGACGCTCTTCCGATCTTACGGRAGGCAGCAG) and R784 (GGAGTTCAGACGTGTGCTCTTCCGATCTTACCAGGGTATCTAATCCT), in 30 cycles with an annealing temperature of 65 °C. To assemble pair-end sequences, the Flash software v1.2.6 [15] was used with an overlap of at least 10-bp between the forward and reverse sequences and by allowing 10% mismatches. Single multiplexing was performed using an in-house 6-bp index, which was added to R784 during a second PCR with 12 cycles and the forward primer (AATGATACGGCGACCACCGAGATCTACACTCTTTCCCTACACGAC) and reverse primer (CAAGCAGAAGACGGCATACGAGAT-index-GTGACTGGAGTTCAGACGTGT). Then, the resulting PCR products were purified and loaded on the Illumina MiSeq cartridge according to the manufacturer’s instructions. The quality of the runs was checked internally using PhiX, and each pair-end sequence was assigned to its sample using the integrated index, with the bcl2fastq Illumina software. The sequences were submitted to the Bioproject database (https://www.ncbi.nlm.nih.gov/bioproject/) with accession number PRJNA701065. Reads were filtered and trimmed for high-quality sequences using the DADA2 package in the R software [16] with the following parameters: maxN = 0, maxEE = 2, truncQ = 2, trimleft = 17. Chimera were removed with the consensus option in DADA2 to obtain the final OTU abundance table. Since no further clustering was applied, the OTU were equivalent to amplicon sequence variants (ASV) in this study. Then, taxonomic annotation was predicted using the assignTaxonomy function of the DADA2 package with the Silva Dataset v132 [17].

To account for differences in sequencing depth across samples, a rarefaction step was applied: for all samples with more than 9000 reads, an equal number of 9000 reads were randomly selected. Sixteen samples had a smaller number of reads and were discarded for later analyses. After this rarefication step, the final abundance table contained 5689 OTU for 588 samples (295 LRFI and 293 HRFI). Finally, following Rothschild et al. [18], OTU in the rarefied table were filtered for more than 1% non-zero values across all sampled animals, which decreased the number of OTU to 2630. Then, a constant value of 1 was added to the rarefied table, which allowed calculation of log values for OTU with zero abundances in the downstream analyses.

### Statistical analyses

#### Estimation of variance components

For each trait, the following four univariate linear models were fitted:

1 $$\mathbf{y}=\mathbf{X}\mathbf{b}+\mathbf{e},$$

2 $$\mathbf{y}=\mathbf{X}\mathbf{b}+{\mathbf{Z}_\mathbf{2}}\mathbf{m}+\mathbf{e},$$

3 $$\mathbf{y}=\mathbf{X}\mathbf{b}+{\mathbf{Z}_\mathbf{1}}\mathbf{a}+\mathbf{e},$$

4 $$\mathbf{y}=\mathbf{X}\mathbf{b}+{\mathbf{Z}_\mathbf{1}}\mathbf{a}+{\mathbf{Z}_\mathbf{2}}\mathbf{m}+\mathbf{e},$$

where $\mathbf{y}$ is the vector of observations of each of the five traits, $\mathbf{b}$ is the vector of fixed effects, $\mathbf{a}$ is the vector of random breeding values, $\mathbf{m}$ is the vector of random microbial values, and $\mathbf{e}$ is the vector of random residuals. $\mathbf{X}$, ${\mathbf{Z}_\mathbf{1}}$ and ${\mathbf{Z}_\mathbf{2}}$ are the incidence matrices for $\mathbf{b}$, $\mathbf{a}$ and $\mathbf{m}$. The distributions assumed for the random effects were $\mathbf{a}\sim N (\mathbf{0}, \mathbf{A}{\upsigma }_{\mathrm{a}}^{2})$, $\mathbf{m}\sim N (\mathbf{0}, \mathbf{M}{\upsigma }_{\mathrm{m}}^{2})$ and $\mathbf{e}\sim N (\mathbf{0},\mathbf{I}{\upsigma }_{\mathrm{e}}^{2})$, where ${\upsigma }_{\mathrm{a}}^{2}$, ${\upsigma }_{\mathrm{m}}^{2}$ and ${\upsigma }_{\mathrm{m}}^{2}$ are the genetic, microbiome and residual variances, respectively; $\mathbf{I}$ denotes the identity matrix; $\mathbf{A}$ is the pedigree relationship matrix based on the 588 animals with microbiota data, plus 6705 ancestors (parents from selection generations G0 to G9 of the lines, plus their ancestors in the shared original population); and $\mathbf{M}$ is the microbial relationship matrix, defined as $\mathbf{M}=\frac{{\mathbf{Z}_\mathbf{3}}{\mathbf{Z}_\mathbf{3}}^{\mathbf{^{\prime}}}}{\mathrm{k}}$, where ${\mathbf{Z}}_{\mathbf{3}}$ is a matrix with dimension $\mathrm{n}\times \mathrm{k}$, with $\mathrm{n}$ being the number of animals with microbiome information and $\mathrm{k}$ the number of OTU [3]. Elements of the ${\mathbf{Z}}_{\mathbf{3}}$ matrix are the standardized individual abundance of each OTU $\mathrm{j}$ for individual $\mathrm{i}$, according to the following equation:

5 $${{\mathrm{z}}_{3}}_{\mathrm{ij}}=\frac{\mathrm{log}\left({\mathrm{P}}_{\mathrm{ij}}\right)-{\overline{\mathrm{log }\left({\mathrm{P}}_{\mathrm{ij}}\right)}}_{\mathrm{j}}}{{\mathrm{sd}(\mathrm{log}\left({\mathrm{P}}_{\mathrm{ij}}\right))}_{\mathrm{j}}},$$

where ${\mathrm{P}}_{\mathrm{ij}}$ is the relative abundance of OTU $\mathrm{j}$ for individual $\mathrm{i}$. The fixed effects fitted in each model were pen size (5 levels), herd of birth (2 levels), sex (3 levels), and contemporary groups (CG, 109 levels, with both lines belonging to each CG). Their significance (p < 0.05) on the five traits was tested in preliminary linear models. The four models were used to evaluate their goodness-of-fit regarding the microbiome effect, using model comparisons between models with and without the microbial values with one degree of freedom, i.e. Model (1) with Model (2) and Model (3) with Model (4).

In addition, to assess if covariance between traits could be due to similar microbiota compositions [5], microbial correlations (${\mathrm{r}}_{\mathrm{m}}$) between the traits were quantified. Bivariate analyses were conducted with Models (2), (3), and (4). In this case, the distributions assumed for the random terms were $\mathbf{a}\sim N (\mathbf{0}, {\mathbf{G}}_\mathbf{0}\otimes \mathbf{A})$, $\mathbf{m}\sim N (\mathbf{0},{\mathbf{R}}_{\mathbf{m}}\otimes \mathbf{M})$ and $\mathbf{e}\sim N (\mathbf{0},{\mathbf{R}}_{\mathbf{e}}\otimes \mathbf{I})$, where $${\mathbf{G}}_\mathbf{0}=\left[\begin{array}{cc}{\upsigma }_{{\mathrm{a}}_{\mathrm{i}}}^{2}& {\upsigma }_{{\mathrm{a}}_{\mathrm{ij}}}\\ {\upsigma }_{{\mathrm{a}}_{\mathrm{ji}}}& {\upsigma }_{{\mathrm{a}}_{\mathrm{j}}}^{2}\end{array}\right]$$ is a $2\times 2$ symmetric (co)variance matrix of genetic effects with the previously defined genetic variances and the genetic correlation ${\mathrm{r}}_{{\mathrm{a}}_{\mathrm{ij}}}=\frac{{\upsigma }_{{\mathrm{a}}_{\mathrm{ij}}}}{{\upsigma }_{{\mathrm{a}}_{\mathrm{i}}}{\upsigma }_{{\mathrm{a}}_{\mathrm{j}}}}$ between each pair of traits $\mathrm{i}$ and $\mathrm{j}$, and similarly $${\mathbf{R}}_{\mathbf{m}}=\left[\begin{array}{cc}{\upsigma }_{{\mathrm{m}}_{\mathrm{i}}}^{2}& {\upsigma }_{{\mathrm{m}}_{\mathrm{ij}}}\\ {\upsigma }_{{\mathrm{m}}_{\mathrm{ji}}}& {\upsigma }_{{\mathrm{m}}_{\mathrm{j}}}^{2}\end{array}\right]$$, with ${\mathrm{r}}_{{\mathrm{m}}_{\mathrm{i}\mathrm{j}}}=\frac{{\upsigma }_{{\mathrm{m}}_{\mathrm{ij}}}}{{\upsigma }_{{\mathrm{m}}_{\mathrm{i}}}{\upsigma }_{{\mathrm{m}}_{\mathrm{j}}}}$, and $${\mathbf{R}}_{\mathbf{e}}=\left[\begin{array}{cc}{\upsigma }_{{\mathrm{e}}_{\mathrm{i}}}^{2}& {\upsigma }_{{\mathrm{e}}_{\mathrm{ij}}}\\ {\upsigma }_{{\mathrm{e}}_{\mathrm{ji}}}& {\upsigma }_{{\mathrm{e}}_{\mathrm{j}}}^{2}\end{array}\right]$$ are $2\times 2$ symmetric (co)variances matrices of microbial and residual effects, respectively.

All models were fitted using the Bayesian framework using the GIBBSF90 software [19]. In total, 100,000 samples were generated to obtain the posterior distributions of the parameters for each model, and a burn-in period of 15,000 samples and thinning interval of 10 were considered. The convergence was verified through visual inspection of trace sample plots. Model comparisons were based on the deviance information criterion (DIC; Spiegelhalter et al. [20]). As a further evaluation of models, correlations of phenotypes adjusted for fixed effects (${\mathbf{y}}^{\mathbf{*}}$) with the sum of solutions obtained for random terms (except residuals) for each individual were calculated and presented as $\mathrm{r}\left({\mathbf{y}}^{\mathbf{*}},\widehat{\mathbf{m}}\right)$ for Model (2), $\mathrm{r}\left({\mathbf{y}}^{\mathbf{*}},\widehat{\mathbf{a}}\right)$ for Model (3) and $\mathrm{r}\left({\mathbf{y}}^{\mathbf{*}},\widehat{\mathbf{a}+\mathbf{m}}\right)$ for Model (4).

### Accuracy of microbiome predictions of phenotypes

To evaluate the accuracy of prediction of the phenotypes using $\widehat{\mathbf{m}}$**,** two scenarios for cross-validation were considered for each of the five traits using Models (2) and (4): the first scenario was designed to run predictions for 50 random animals in 20 successive replicates, and the second scenario was designed to run predictions for the animals of each of the 14 CG, successively. The objective with these designs was to evaluate the effect of the presence of contemporary animals on the prediction accuracy.

The part-whole linear regression (LR) method of Legarra and Reverter [21] was used to quantify prediction accuracies. This is an alternative to the conventional calculation of accuracy based on correlations with adjusted phenotypes, which can be affected by the limited accuracy of the computation of adjusted phenotypes. For the LR method, the phenotypes of the animals to be predicted were set to missing for each trait and their $\widehat{\mathbf{m}}$ were predicted (${\widehat{\mathbf{m}}}_{\mathbf{p}}$, i.e. prediction using a partial dataset) with Models (2) and (4). For each replicate, prediction accuracy for each trait was evaluated based on the correlation between ${\widehat{\mathbf{m}}}_{\mathbf{p}}$ and predicted $\widehat{\mathbf{m}}$ using the full dataset (${\widehat{\mathbf{m}}}_{\mathbf{w}}$, i.e., prediction using the whole dataset) with the same model. The final criterion was the average of the correlations across replicates $(\overline{\mathrm{r }\left({\widehat{\mathbf{m}}}_{\mathbf{p}},{\widehat{\mathbf{m}}}_{\mathbf{w}}\right)})$. Similarly, to evaluate the accuracy of microbiome predictions of phenotypes with Model (4), the average of part-whole correlations for the sum of predicted breeding values and microbial value was calculated, i.e. $\overline{\mathrm{r }\left({(\widehat{\mathbf{a}+\mathbf{m}})}_{\mathbf{p}},{(\widehat{\mathbf{a}+\mathbf{m}})}_{\mathbf{w}}\right)}$.

### Microbiome-wide association studies (MWAS)

The objective of the MWAS was to identify OTU that have significant associations with the phenotype of each of the five traits. Two approaches were used.

#### Single-OTU regression analysis

Single-OTU regression analyses were applied to test the effect of the 2630 OTU, one at a time, and obtain the associated p-value, which is the most common approach [10]. The model used for these analyses was the same as Model (3) except that a specific OTU was added as a fixed covariate. The model was fitted using the best linear unbiased prediction (BLUP) method of the AIREMLF90 software [19]. The p-value of the estimate of the regression coefficient for the fitted OTU covariate was obtained by converting the estimate and its standard error to a Z-score and applying a Chi-squared test.

#### Back-solving BLUP solutions

An alternative approach is the back-solving of predicted $\widehat{\mathbf{m}}$ from Model (4) to obtain the estimated effect of each OTU on the trait phenotypes. This approach is similar to those described by Stranden and Garrick [22] and Gualdron-Duarte et al. [23] to obtain estimates and prediction error variances for single nucleotide polymorphisms (SNPs) from estimated breeding values using genomic-BLUP. Such back-solving is often used for SNP genome-wide association studies. It has only recently been proposed for microbiota analyses [11], however, the corresponding p-values of the estimates have not been reported. The back-solving approach and calculation of the p-values were conducted as described in the following.

Estimates for the effect of each OTU $\widehat{(\mathbf{O}\mathbf{T}\mathbf{U})}$ can be obtained if the assumptions of ${\upsigma }_{\mathrm{OTU}}^{2}={\upsigma }_{\mathrm{m}}^{2}/\mathrm{k}$ and $\mathbf{D}=\mathbf{I}{\upsigma }_{\mathrm{m}}^{2}/\mathrm{k}$ hold, and thus:

6 $${\mathbf{Z}}_{\mathbf{3}}\mathbf{D}{\mathbf{Z}}_{\mathbf{3}}^{\mathbf{\prime}}=\mathbf{M}{\upsigma }_{\mathrm{m}}^{2}.$$

Therefore, estimates for the effects of OTU given the $\widehat{\mathbf{m}}$ can be achieved as follows [22]:

7 $$\mathrm{E}(\mathbf{O}\mathbf{T}\mathbf{U})=\widehat{\mathbf{O}\mathbf{T}\mathbf{U}}={\mathbf{D}}{\mathbf{Z}_\mathbf{3}}^{\mathbf{\prime}}\left({\mathbf{Z}_\mathbf{3}}{\mathbf{D}}{\mathbf{Z}_\mathbf{3}}^{\mathbf{\prime}}\right)^{\mathbf{-1}}\widehat{\mathbf{m}}=\frac{1}{\mathrm{k}}{\mathbf{Z}_\mathbf{3}}^{\mathbf{\prime}}{\mathbf{M}}^{\mathbf{-1}}\widehat{\mathbf{m}}.$$

And the variance of the OTU estimates is defined as in [23]:

8 $$\mathrm{var}\left(\widehat{\mathbf{O}\mathbf{T}\mathbf{U}}\right)=\mathrm{var}\left(\frac{1}{\mathrm{k}}{\mathbf{Z}_\mathbf{3}}^{\mathbf{\prime}}{\mathbf{M}}^{\mathbf{-1}}\widehat{\mathbf{m}}\right)=\frac{1}{\mathrm{k}}{\mathbf{Z}_\mathbf{3}}^{\mathbf{\prime}}{\mathbf{M}}^{\mathbf{-1}}\mathrm{var}\left(\widehat{\mathbf{m}}\right){\mathbf{M}}^{\mathbf{-1}}{\mathbf{Z}}_{3}\frac{1}{\mathrm{k}}.$$

The predictor error variance (PEV) of $\widehat{\mathbf{m}}$ is equal to:

9 $$\mathbf{P}\mathbf{E}\mathbf{V}\left(\widehat{\mathbf{m}}\right)=\mathrm{var}\left(\mathbf{m}-\widehat{\mathbf{m}}\right)=\mathrm{var}\left(\mathbf{m}\right)-\mathrm{var}(\widehat{\mathbf{m}})={\mathbf{C}}^{\mathbf{m}\mathbf{m}}{\upsigma }_{\mathrm{e}}^{2}$$

Therefore,

10 $$\mathrm{var}\left(\widehat{\mathbf{m}}\right)=\mathrm{var}\left(\mathbf{m}\right)-{\mathbf{C}}^{\mathbf{m}\mathbf{m}}{\upsigma }_{\mathrm{e}}^{2}=\mathbf{M}{\upsigma }_{\mathrm{m}}^{2}-{\mathbf{C}}^{\mathbf{m}\mathbf{m}}{\upsigma }_{\mathrm{e}}^{2},$$

where ${\mathbf{C}}^{\mathbf{m}\mathbf{m}}$ are the diagonal elements of the sub-matrix corresponding to the random microbial values from the inverse of coefficient matrix of the mixed model equations.

Finally:

11 $$\mathrm{var}\left(\widehat{\mathbf{O}\mathbf{T}\mathbf{U}}\right)=\frac{1}{\mathrm{k}}{\mathbf{Z}_\mathbf{3}}^{\mathbf{{\prime}}}{\mathbf{M}}^{\mathbf{-1}}\left(\mathbf{M}{\upsigma }_{\mathrm{m}}^{2}-{\mathbf{C}}^{\mathbf{m}\mathbf{m}}{\upsigma }_{\mathrm{e}}^{2}\right){\mathbf{M}}^{\mathbf{-1}}{\mathbf{Z}_\mathbf{3}}\frac{1}{\mathrm{k}}.$$

Then, the Z-score for the estimate for each OTU $\mathrm{j}$ can be obtained from the diagonal elements of $\mathrm{var}\left(\widehat{\mathbf{O}\mathbf{T}\mathbf{U}}\right)$ as:

12 $${\mathrm{Z}-\mathrm{score}}_{\mathrm{j}}=\frac{\widehat{\mathrm{OTU_j}}}{\sqrt{\mathrm{var}\left(\widehat{\mathrm{OTU_j}}\right)}}.$$

The corresponding p-values can then be calculated by applying a Chi-square test to these Z-scores.

The back-solving method was run using a local script to construct and solve the mixed model equations based on the variance component estimates of Model (4) for each trait. It should be noted that both the single-OTU regression and the back-solving approach accounted for additive genetic effects in order to obtain comparable results.

### Significance thresholds for MWAS

To obtain significance thresholds for the MWAS to control the family-wise type I error rate while accounting for multiple testing using non-independent variables, a principal component analysis (PCA) was applied to the correlation matrix of OTU $({\mathbf{Z}_\mathbf{3}}^{\mathbf{{\prime}}}{\mathbf{Z}_\mathbf{3}}_{\mathbf{2630}\times\mathbf{2630}})$ to estimate the number of independent tests, as proposed by Gao et al. [24]. The PCA showed that 428 eigenvalues captured 99.5% of the variability in the correlation matrix. Based on this, thresholds for significance and suggestive significance at 5% (−log10(0.05/428)) and 10% (− log10(0.10/428)) family-wise type I error rates were used to test the significance of the effects of OTU.

## Results

### Estimation of variance components

The results of univariate analyses of the five studied traits with the four models are in Table 2, as posterior means ± posterior 95% confidence intervals of each variance component. Comparisons of the DIC values showed a consistent improvement of the fit from Model (1) to Model (4), which had the smallest DIC for all traits. This goodness-of-fit of Model (4) was confirmed by its higher $\mathrm{r}({\mathbf{y}}^{\mathbf{*}},\widehat{\mathbf{a}+\mathbf{m}})$, compared to the correlations between ${\mathbf{y}}^{\mathbf{*}}$ and the predictions from Models (2) and (3) (Table 3). The posterior means for heritability were moderate for all traits and ranged from 0.31 ± 0.13 for FCR to 0.51 ± 0.10 for BFT, with no difference between estimates from Models (3) and (4). The microbiome variances obtained with Models (2) and (4) showed a substantial contribution to the phenotypic variance of feed efficiency traits, with posterior means for ${\mathrm{m}}^{2}$ of, respectively, 0.12 ± 0.09 and 0.11 ± 0.09 for RFI and of 0.22 ± 0.11 and 0.20 ± 0.11 for FCR. In contrast, the phenotypic variances of DFI, BFT and ADG were less influenced by the microbiome variance. The posterior means for ${\mathrm{m}}^{2}$ were lower than 0.06 ± 0.06 for DFI and ADG for Models (2) and (4), and for BFT it ranged from 0.11 ± 0.06 with Model (2) to 0.02 ± 0.03 with Model (4), i.e. mainly close to zero. All traits showed lower posterior means for ${\mathrm{m}}^{2}$ than for ${\mathrm{h}}^{2}$.

**Table 2:** Posterior means (± posterior standard deviation) of variance components, heritability, and microbiability of the production traits using the four models, and the deviance information criterion (DIC) of each model and trait

Trait	Model	${\upsigma }_{\mathrm{g}}^{2}$	${\upsigma }_{\mathrm{m}}^{2}$	${\upsigma }_{\mathrm{e}}^{2}$	${\upsigma }_{\mathrm{p}}^{2}$	${\mathrm{h}}^{2}$	${\mathrm{m}}^{2}$	DIC
RFI	(1)	–	–	0.020 ± 0.001	0.020 ± 0.001	–	–	− 343032380251
	(2)	–	0.002 ± 0.002	0.017 ± 0.002	0.020 ± 0.001	–	0.12 ± 0.09	− 376715009798
	(3)	0.006 ± 0.002	–	0.014 ± 0.002	0.020 ± 0.001	0.32 ± 0.10	–	− 481592517646
	(4)	0.006 ± 0.002	0.002 ± 0.002	0.012 ± 0.002	0.020 ± 0.001	0.30 ± 0.10	0.11 ± 0.09	− 540693245186
FCR	(1)	–	–	0.062 ± 0.004	0.062 ± 0.004	–	–	− 64257520104
	(2)	–	0.014 ± 0.008	0.051 ± 0.007	0.065 ± 0.005	–	0.22 ± 0.11	− 78170373902
	(3)	0.024 ± 0.010	–	0.043 ± 0.007	0.067 ± 0.005	0.35 ± 0.13	–	− 93388582161
	(4)	0.022 ± 0.010	0.014 ± 0.008	0.032 ± 0.009	0.070 ± 0.006	0.31 ± 0.13	0.20 ± 0.11	− 122763803675
DFI	(1)	–	–	0.051 ± 0.003	0.051 ± 0.003	–	–	− 89420640342
	(2)	–	0.003 ± 0.003	0.050 ± 0.004	0.052 ± 0.003	–	0.06 ± 0.06	− 93351683359
	(3)	–	–	0.030 ± 0.006	0.056 ± 0.005	0.50 ± 0.13	–	− 167531111339
	(4)	0.030 ± 0.010	0.002 ± 0.002	0.030 ± 0.007	0.060 ± 0.005	0.48 ± 0.14	0.04 ± 0.03	− 173089799612
ADG	(1)	–	–	0.0051 ± 0.0003	0.0051 ± 0.0003	–	–	− 255536305168
	(2)	–	0.0002 ± 0.0003	0.0050 ± 0.0004	0.0051 ± 0.0003	–	0.05 ± 0.05	− 265759148148
	(3)	0.0024 ± 0.0009	–	0.0030 ± 0.0006	0.0054 ± 0.0005	0.45 ± 0.13	–	− 440920106295
	(4)	0.0030 ± 0.0008	0.0001 ± 0.0002	0.0030 ± 0.0006	0.0055 ± 0.0005	0.47 ± 0.12	0.03 ± 0.03	− 471061077561
BFT	(1)	–	–	8.854 ± 0.570	8.854 ± 0.570	–	–	− 525486682
	(2)	–	0.100 ± 0.610	8.057 ± 0.680	9.055 ± 0.604	–	0.11 ± 0.06	− 575243859
	(3)	4.754 ± 1.301	–	4.695 ± 0.872	9.450 ± 0.750	0.50 ± 0.11	–	− 999015096
	(4)	4.980 ± 1.280	0.228 ± 0.314	4.424 ± 0.878	9.636 ± 0.760	0.51 ± 0.10	0.02 ± 0.03	− 1071710845

${\upsigma }_{\mathrm{g}}^{2}$: genetic variance, ${\upsigma }_{\mathrm{m}}^{2}$: microbiome variance, ${\upsigma }_{\mathrm{e}}^{2}$: residual variance, ${\upsigma }_{\mathrm{p}}^{2}$: phenotypic variance, ${\mathrm{h}}^{2}$: heritability, ${\mathrm{m}}^{2}$: microbiability

*RFI* residual feed intake (kg/day), *FCR* feed conversion ratio (kg/kg), *DFI* daily feed intake (kg/day), *ADG* average daily gain (kg/day), *BFT* backfat thickness (mm)

**Table 3:** Pearson correlations of estimated breeding values ($\widehat{\mathrm{a}}$) and estimated microbial values ($\widehat{\mathrm{m}}$) with the adjusted phenotypes of the production traits (${{\mathrm{y}}^{*}}$) for the three models

Trait	Model		
	(2)	(3)	(4)
	$\mathrm{r}({\mathrm{y}}^{*},\widehat{\mathrm{m}})$	$\mathrm{r}({\mathrm{y}}^{*},\widehat{\mathrm{a}})$	$\mathrm{r}({\mathrm{y}}^{*},\widehat{\mathrm{a}+\mathrm{m}})$
RFI	0.79	0.79	0.88
FCR	0.81	0.79	0.88
DFI	0.68	0.76	0.84
ADG	0.61	0.75	0.80
BFT	0.62	0.88	0.92

*RFI* residual feed intake, *FCR* feed conversion ratio, *DFI* daily feed intake, *ADG* average daily gain, *BFT* backfat thickness

The results of the bivariate analyses for the studied traits with Models (2), (3), and (4) are in Table 4. Posterior means for ${\mathrm{h}}^{2}$ and ${\mathrm{m}}^{2}$ of the traits in these analyses were similar to those obtained with the univariate analyses. Given the posterior standard deviations, estimates of Models (2) and (3) were not different from the corresponding estimates of Model (4), except for the estimate for ${\mathrm{r}}_{\mathrm{m}}$ between ADG and BFT. Estimates of ${\mathrm{r}}_{\mathrm{m}}$ from Model (4) ranged from − 0.54 ± 0.60 for RFI and ADG to 0.96 ± 0.11 for ADG and BFT. Except for the estimate of ${\mathrm{r}}_{\mathrm{m}}$ between RFI and FCR (0.70 ± 0.34), all other estimates had high standard errors given the low microbiability estimates of the traits. Estimates of genetic correlations, ${\mathrm{r}}_{\mathrm{a}}$, were very similar for Models (3) and (4).

**Table 4:** Posterior means (± posterior standard deviation) of the heritability, microbiability obtained from bivariate analyses between production traits with models (2), (3) and (4)

Model	Trait 1	Trait 2	${\mathrm{h}}_{\mathrm{T}1}^{2}$	${\mathrm{h}}_{\mathrm{T}2}^{2}$	${\mathrm{m}}_{\mathrm{T}1}^{2}$	${\mathrm{m}}_{\mathrm{T}2}^{2}$	${\mathrm{r}}_{\mathrm{a}12}$	${\mathrm{r}}_{\mathrm{m}12}$
(2)	RFI	FCR	–	–	0.12 ± 0.13	0.25 ± 0.09	–	0.75 ± 0.33
		DFI	–	–	0.26 ± 0.11	0.04 ± 0.05	–	0.87 ± 0.33
		ADG	–	–	0.12 ± 0.10	0.09 ± 0.07	–	− 0.87 ± 0.39
		BFT	–	–	0.23 ± 0.10	0.01 ± 0.00	–	− 1.00 ± NE
	FCR	DFI	–	–	0.23 ± 0.12	0.12 ± 0.07	–	0.66 ± 0.46
		ADG	–	–	0.17 ± 0.11	0.13 ± 0.07	–	− 0.94 ± 0.13
		BFT	–	–	0.24 ± 0.13	0.06 ± 0.07	–	0.76 ± 0.40
	DFI	ADG	–	–	0.03 ± 0.04	0.13 ± 0.07	–	− 0.38 ± 0.66
		BFT	–	–	0.06 ± 0.04	0.16 ± 0.07	–	0.88 ± 0.32
	ADG	BFT	–	–	0.04 ± 0.05	0.14 ± 0.06	–	− 0.28 ± 0.66
(3)	RFI	FCR	0.35 ± 0.12	0.40 ± 0.12	–	–	0.64 ± 0.16	–
		DFI	0.31 ± 0.10	0.51 ± 0.14	–	–	0.49 ± 0.22	–
		ADG	0.33 ± 0.10	0.52 ± 0.13	–	–	− 0.01 ± 0.24	–
		BFT	0.30 ± 0.09	0.53 ± 0.10	–	–	0.002 ± 0.20	–
	FCR	DFI	0.33 ± 0.15	0.52 ± 0.14	–	–	0.50 ± 0.26	–
		ADG	0.39 ± 0.13	0.47 ± 0.14	–	–	− 0.23 ± 0.24	–
		BFT	0.34 ± 0.12	0.51 ± 0.12	–	–	0.59 ± 0.20	–
	DFI	ADG	0.50 ± 0.12	0.49 ± 0.13	–	–	0.64 ± 0.16	–
		BFT	0.52 ± 0.14	0.55 ± 0.12	–	–	0.65 ± 0.14	–
	ADG	BFT	0.54 ± 0.15	0.53 ± 0.11	–	–	0.30 ± 0.19	–
(4)	RFI	FCR	0.35 ± 0.11	0.38 ± 0.13	0.16 ± 0.10	0.23 ± 0.10	0.66 ± 0.16	0.70 ± 0.34
		DFI	0.29 ± 0.09	0.54 ± 0.13	0.18 ± 0.12	0.06 ± 0.04	0.63 ± 0.17	0.71 ± 0.47
		ADG	0.33 ± 0.10	0.51 ± 0.13	0.09 ± 0.08	0.10 ± 0.05	0.00 ± 0.27	− 0.54 ± 0.60
		BFT	0.29 ± 0.10	0.52 ± 0.11	0.17 ± 0.08	0.05 ± 0.03	0.02 ± 0.27	− 1.00 ± NE
	FCR	DFI	0.32 ± 0.12	0.52 ± 0.12	0.28 ± 0.10	0.08 ± 0.03	0.44 ± 0.25	0.99 ± NE
		ADG	0.38 ± 0.13	0.51 ± 0.13	0.22 ± 0.10	0.11 ± 0.05	− 0.25 ± 0.21	− 0.91 ± 0.18
		BFT	0.34 ± 0.11	0.49 ± 0.10	0.23 ± 0.09	0.05 ± 0.04	0.52 ± 0.20	0.40 ± 0.64
	DFI	ADG	0.48 ± 0.13	0.49 ± 0.12	0.04 ± 0.04	0.12 ± 0.06	0.62 ± 0.16	− 0.37 ± 0.56
		BFT	0.50 ± 0.13	0.51 ± 0.10	0.04 ± 0.04	0.06 ± 0.04	0.61 ± 0.15	0.50 ± 0.58
	ADG	BFT	0.49 ± 0.14	0.50 ± 0.11	0.04 ± 0.05	0.06 ± 0.05	0.30 ± 0.20	0.96 ± 0.11

${\mathrm{h}}_{\mathrm{T}1}^{2}$: heritability of first trait, ${\mathrm{h}}_{\mathrm{T}2}^{2}$: heritability of second trait, ${\mathrm{m}}_{\mathrm{T}1}^{2}$: microbiability of first trait, ${\mathrm{m}}_{\mathrm{T}2}^{2}$: microbiability of second trait, ${\mathrm{r}}_{\mathrm{a}12}$: genetic correlation, ${\mathrm{r}}_{\mathrm{m}12}$: microbial correlation, NE: not estimable

*RFI* residual feed intake, *FCR* feed conversion ratio, *DFI* daily feed intake, *ADG* average daily gain, *BFT* backfat thickness

### Accuracy of microbiome predictions of phenotypes

**Fig. 1:**
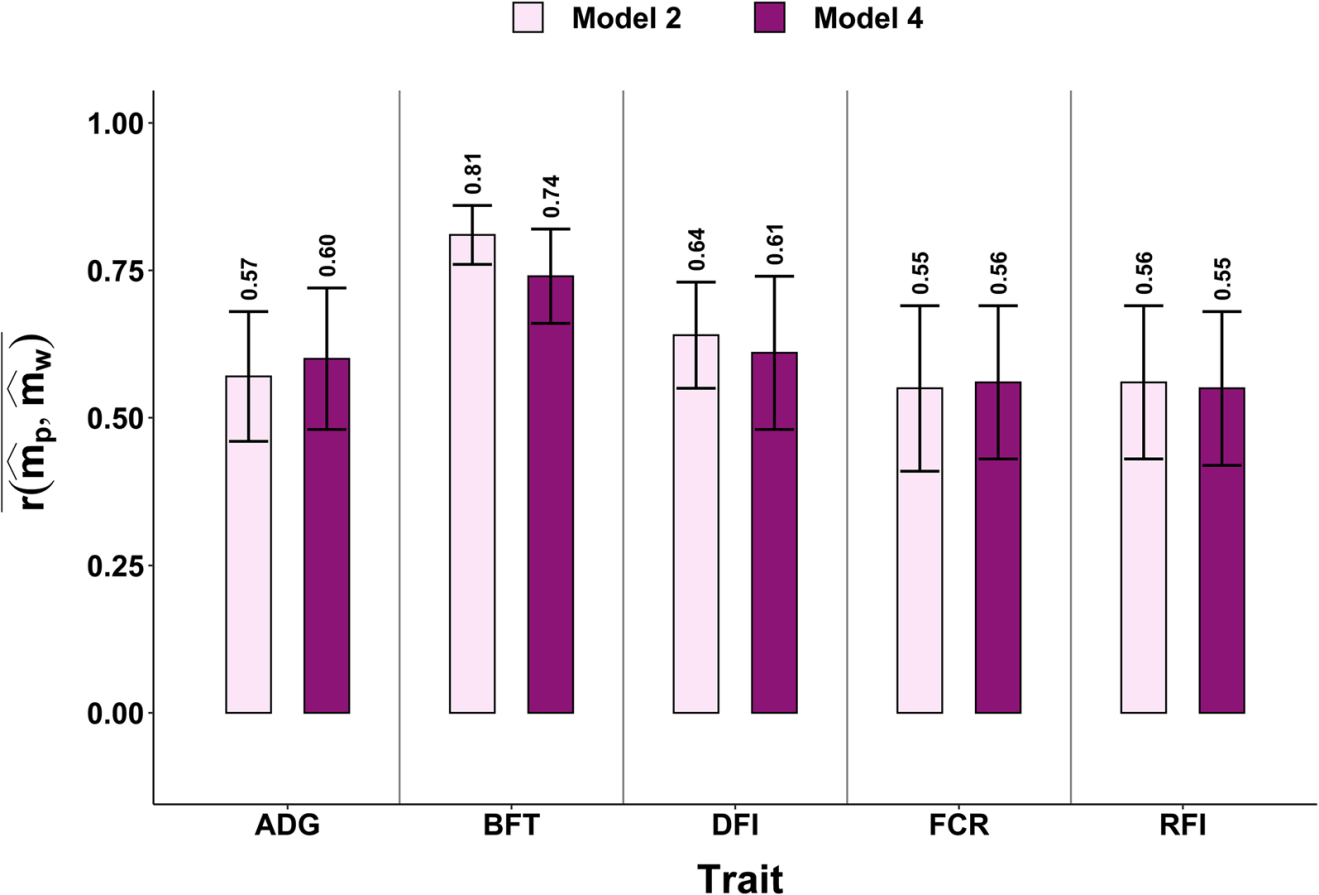
Average correlations between ${\widehat{\mathbf{m}}}_{\mathbf{p}}$ ($\widehat{\mathbf{m}}$ using partial datasets) and ${\widehat{\mathbf{m}}}_{\mathbf{w}}$ ($\widehat{\mathbf{m}}$ using the whole dataset) for 50 random animals in 20 replicates, and their SD as error bars. *ADG* average daily gain, *BFT* backfat thickness, *DFI* daily feed intake, *FCR* feed conversion ratio, *RFI* residual feed intake, *EMV* estimated microbiota values

The $\overline{\mathrm{r }\left({\widehat{\mathbf{m}}}_{\mathbf{p}},{\widehat{\mathbf{m}}}_{\mathbf{w}}\right)}$ for 50 random animals in 20 replicates for the five traits are shown in Fig. 1 and the $\overline{\mathrm{r }\left({\widehat{\mathbf{m}}}_{\mathbf{p}},{\widehat{\mathbf{m}}}_{\mathbf{w}}\right)}$ for CG are given in Fig. S1 (see Additional file 1: Fig. S1). The average correlations ± SD ranged from 0.55 ± 0.14 to 0.81 ± 0.05 for the random design and from 0.39 ± 0.23 to 0.70 ± 0.15 for the CG design, depending on the trait. The $\overline{\mathrm{r }\left({\widehat{\mathbf{m}}}_{\mathbf{p}},{\widehat{\mathbf{m}}}_{\mathbf{w}}\right)}$ were systematically slightly higher with the random design than with the CG design for both Models (2) and (4), but differences were within the range of the SE. Finally, including the genetic effect in Model (4) did not improve prediction accuracy of $\widehat{\mathbf{m}}$. With the random design, the $\overline{\mathrm{r }\left({\widehat{\mathbf{m}}}_{\mathbf{p}},{\widehat{\mathbf{m}}}_{\mathbf{w}}\right)}$ for BFT was higher than for the other four traits, which had corresponding accuracies around 0.60. With the CG design, the $\overline{\mathrm{r }\left({\widehat{\mathbf{m}}}_{\mathbf{p}},{\widehat{\mathbf{m}}}_{\mathbf{w}}\right)}$ was slightly lower for FCR and RFI than for ADG, BFT and DFI.

**Fig. 2:**
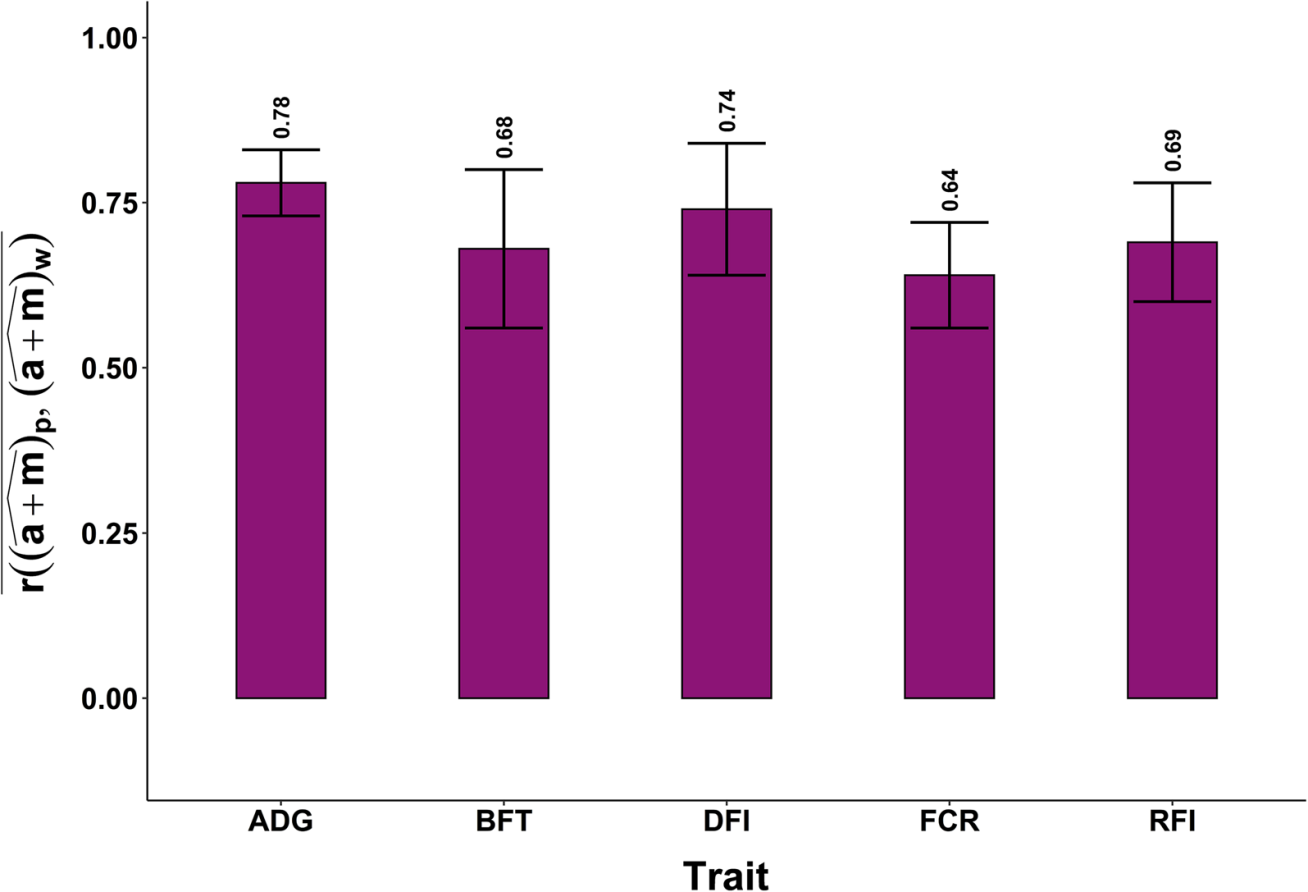
Average correlations between ${(\widehat{\mathbf{a}+\mathbf{m}})}_{\mathbf{p}}$ and ${(\widehat{\mathbf{a}+\mathbf{m}})}_{\mathbf{w}}$ for 50 random animals in 20 replicates, and their SD as error bars. *ADG* average daily gain, *BFT* backfat thickness, *DFI* daily feed intake, *FCR* feed conversion ratio, *RFI* residual feed intake

Estimates of $\overline{\mathrm{r }\left({(\widehat{\mathbf{a}+\mathbf{m}})}_{\mathbf{p}},{(\widehat{\mathbf{a}+\mathbf{m}})}_{\mathbf{w}}\right)}$ obtained with Model (4) ranged from 0.68 ± 0.12 to 0.78 ± 0.05 with the random design (Fig. 2) and from 0.60 ± 0.19 to 0.75 ± 0.10 with the CG design (see Additional file 1: Fig. S2). With both designs, the highest value was obtained for ADG and the lowest value for FCR. Although not significantly different, these values were higher than the corresponding $\overline{\mathrm{r }\left({\widehat{\mathbf{m}}}_{\mathbf{p}},{\widehat{\mathbf{m}}}_{\mathbf{w}}\right)}$ values for all traits, except for BFT with the random design, for which adding $\widehat{\mathbf{a}}$ resulted in slightly lower accuracies (from 0.74 to 0.68).

### Microbiome-wide association studies

**Fig. 3:**
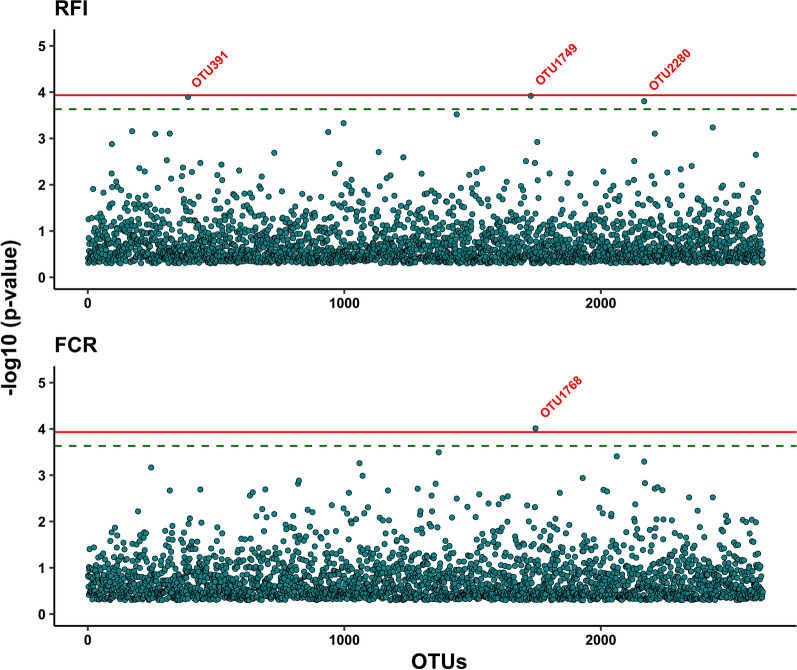
Results of microbiome wide association analyses using single-OTU regression method between operational taxonomic units and residual feed intake (RFI) and feed conversion ratio (FCR). In the plots, the solid and dashed lines represent significance and suggestive significance at 5 and 10% family-wise type I error rates, respectively

**Fig. 4:**
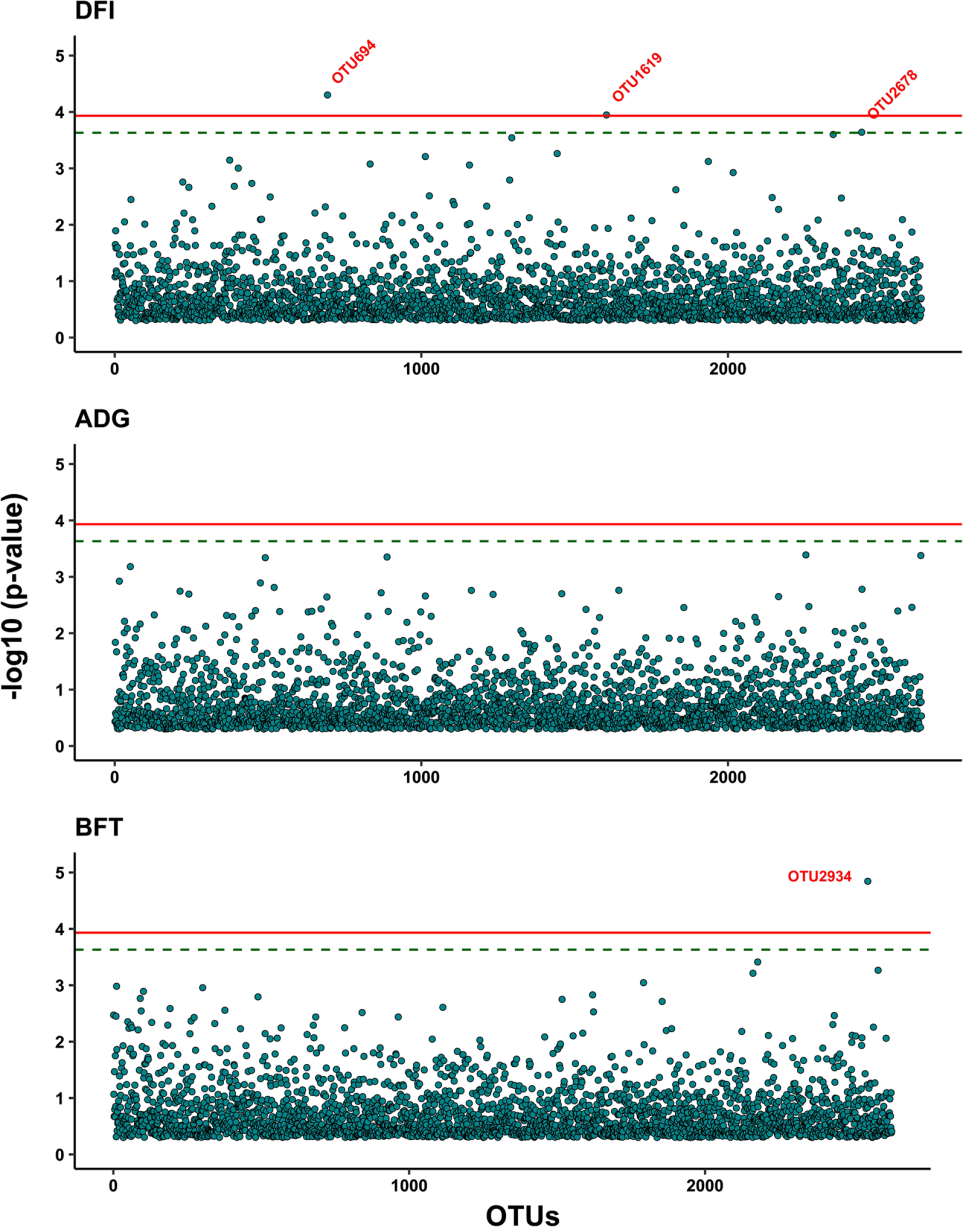
Results of microbiome wide association analyses using single-OTU regression method between operational taxonomic units and daily feed intake (DFI), average daily gain (ADG) and back fat thickness (BFT). In the plots, the solid and dashed lines represent significance and suggestive significance at 5 and 10% family-wise type I error rates, respectively

The results of MWAS with single-OTU regression are shown in Fig. 3 (RFI and FCR) and Fig. 4 (DFI, ADG, and BFT), and those from the back-solving approach are given in Figure S3 (see Additional file 1: Fig. S3). The two approaches used for MWAS resulted in similar estimates, but the single-regression method showed slightly higher significance levels, although Rubio et al. [25] have demonstrated the theoretical equivalence between these two approaches. There were no common significant or suggestive OTU between the traits. For RFI, the single-OTU regression resulted in three suggestive OTU (OTU391, OTU1749, and OTU2280), while the back-solving method resulted in one significant (OTU391) and one suggestive OTU (OTU1749). Both approaches identified one significant OTU for FCR (OTU1768) and one for BFT (OTU2934). For DFI, the single-OTU regression identified two significant (OTU694 and OTU1619) and one suggestive OTU (OTU2678), while the back-solving method resulted in one significant (OTU694) and one suggestive OTU (OTU1619). For ADG, none of the methods detected significant or suggestive OTU.

The eight OTU with significant or suggestive associations with at least one of the five traits belonged to the *Streptococcaceae* (1 OTU), *Prevotellaceae* (3 OTU), *Ruminococcaceae* (3 OTU), and *Lachnospiraceae* (1 OTU) families (Table 5). From these, the six OTU with an identified genus belonged to different genera. All these genera had more than 85% of zero count, and only OTU391, associated with RFI, had on average more than 10 counts per sample when it was present. The estimated regression coefficients (Table 3) indicated that higher abundances of this OTU and of OTU1749 were associated with greater efficiency (reduced RFI). Higher abundances of OTU2280, OTU1768, and OTU2934 were associated with greater RFI, FCR, and BFT, respectively, while an increase in abundance in each of the detected OTU for DFI was associated with reduced feed intake.

**Table 5:** Taxonomy, average counts per sample and estimates of regression coefficients from single-OTU regression for the OTU with significant or suggestive associations with the production traits

OTU	Phylum	Class	Order	Family	Genus	%Zero	Average counts	Average counts for non-zero samples	Associated trait	Regression coefficient
OTU391	*Firmicutes*	*Bacilli*	*Lactobacillales*	*Streptococcaceae*	*Streptococcus*	92.7	2.33	31.88	RFI	− 0.09
OTU1749	*Bacteroidetes*	*Bacteroidia*	*Bacteroidales*	*Prevotellaceae*	*Prevotella_9*	98.8	0.11	9.00		− 0.10
OTU2280	*Firmicutes*	*Clostridia*	*Clostridiales*	*Ruminococcaceae*	*unknown*	97.8	0.06	2.70		0.11
OTU1768	*Firmicutes*	*Clostridia*	*Clostridiales*	*Ruminococcaceae*	*Ruminococcus 1*	98.5	0.14	9.22	FCR	0.17
OTU694	*Firmicutes*	*Clostridia*	*Clostridiales*	*Ruminococcaceae*	*Ruminococcaceae UCG-014*	85.0	1.00	6.67	DFI	− 0.06
OTU1619	*Bacteroidetes*	*Bacteroidia*	*Bacteroidales*	*Prevotellaceae*	*Alloprevotella*	94.9	0.19	3.70		− 0.14
OTU2678	*Firmicutes*	*Clostridia*	*Clostridiales*	*Lachnospiraceae*	*XBB1006*	98.3	0.03	1.90		− 0.25
OTU2934	*Bacteroidetes*	*Bacteroidia*	*Bacteroidales*	*Prevotellaceae*	*unknown*	98.6	0.02	1.50	BFT	4.46

*RFI* residual feed intake (kg/day), *FCR* feed conversion ratio (kg/kg), *DFI* daily feed intake (kg/day), *ADG* average daily gain (kg/day), *BFT* backfat thickness (mm)

## Discussion

### Estimates of variance components

Previous studies in pigs revealed that the abundance of some components of the microbial community are heritable, and that heritability estimates vary at different stages of pig growth [3, 10, 26], which would provide a lever to select these GIT microbial components across generations. Such a stability of the microbial community across generations could be used to orientate the phenotypes of the host animals. Therefore, in the present study, gut microbial information of two divergent pig lines was fitted into linear animal mixed models to quantify the proportion of the phenotypic variance of feed efficiency and other performance traits captured by this new component. The analyses showed that the microbial variance was substantial for traits related to feed efficiency. The estimate of ${\mathrm{m}}^{2}$ obtained for RFI in our study was lower than the value (0.45 ± 0.15) reported by Weishaar et al. [26]. For FCR, the estimates of ${\mathrm{m}}^{2}$ were within the range of those reported by Camarinha-Silva et al. [3] (0.21 ± 0.14) and by Weishaar et al. [27] (0.13 ± 0.10). For the other performance traits, low estimates of ${\mathrm{m}}^{2}$ were obtained, in spite of the lower DIC obtained for Models (2) and (4) than for Models (1) and (3), respectively. In their study, Camarinha-Silva et al. [3] reported a non-significant ${\mathrm{m}}^{2}$ estimate of 0.16 ± 0.10 for feed intake, which was higher than our estimates of ${\mathrm{m}}^{2}$ with Models (2) and (4) for DFI, but not significantly different. Camarinha-Silva et al. [3] and Weishaar et al. [27] reported moderate estimates of ${\mathrm{m}}^{2}$ of 0.28 ± 0.13 and 0.24 ± 0.11, respectively, for ADG, which were higher than our estimates for this trait. Khanal et al. [5], in a study on the microbiability of meat quality and carcass composition traits in swine, found that the estimate of ${\mathrm{m}}^{2}$ for backfat depth increased with age at sampling, i.e. 0.01 ± 0.02 at weaning, 0.12 + 0.04 at mid-test, and 0.25 ± 0.04 at off-test. Our estimates of ${\mathrm{m}}^{2}$ for BFT with the full Model (4) are comparable with their value at weaning, although the sampling time in our study is equivalent to their mid-test sampling. Compared to our study, these three previous studies used different genetic types (Piétrain or commercial crossbreds), sample sizes, and times of collection, which may explain part of the observed differences. In addition, differences in the bioinformatics processing and clustering rules of the sequences to obtain OTU tables are a source of heterogeneity between studies. The aim of our choice to work with ASV, i.e. without a clustering step, was to reduce part of this heterogeneity and to facilitate comparisons between studies [28].

To our knowledge, except for one study on meat quality and carcass composition traits [5] that considered carcass ADG and fat depth, microbial correlations between the traits studied here have not been reported in pigs. The positive high estimate of ${\mathrm{r}}_{\mathrm{m}}$ between RFI and FCR suggests that a common microbial community influences both traits. Khanal et al. [5] observed a decrease in some estimates of genomic correlations between meat quality and carcass traits in pigs when a microbial correlation was included in the models, and argued that the genomic correlations between traits usually estimated could be partially due to correlations between the gut microbiota composition of animals. However, given that we had previously observed significant genetic correlations between the microbial components and the studied traits [12], the reverse hypothesis may be also relevant, i.e. that part of the ${\mathrm{r}}_{\mathrm{m}}$ between the traits may be due to the high genetic correlations between the traits. Nevertheless, none of these phenomena were actually observed in the current study, since the posterior means of the correlations were quite stable across models, which suggests that estimates of ${\mathrm{r}}_{\mathrm{a}}$ and ${\mathrm{r}}_{\mathrm{m}}$ between the traits do not depend on each other.

### Accuracy of microbiome predictions of phenotypes

Our objective was to investigate if inclusion of microbial information collected at mid-test in the BLUP models can improve phenotype predictions of animals based on $\widehat{\mathbf{m}}$ only (Model 2) or $\widehat{\mathbf{a}+\mathbf{m}}$ (Model 4). Using only the microbiome information, prediction accuracies were slightly lower than when both the microbiome and genetic information were used (higher $\overline{\mathrm{r }\left({(\widehat{\mathbf{a}+\mathbf{m}})}_{\mathbf{p}},{(\widehat{\mathbf{a}+\mathbf{m}})}_{\mathbf{w}}\right)}$ than $\overline{\mathrm{r }\left({\widehat{\mathbf{m}}}_{\mathbf{p}},{\widehat{\mathbf{m}}}_{\mathbf{w}}\right)}$). In details, the prediction accuracies of the microbiome part were very similar with Models 2 and 4, suggesting that adding the genetic effect in Model 4 provided additional accuracy of prediction of phenotypes by capturing new information rather than by better identifying the genetic versus microbiome information in the models. Overall, the relatively high values of the $\overline{\mathrm{r }\left({(\widehat{\mathbf{a}+\mathbf{m}})}_{\mathbf{p}},{(\widehat{\mathbf{a}+\mathbf{m}})}_{\mathbf{w}}\right)}$ suggest that phenotypes of animals for the studied traits can be predicted with a reasonable accuracy with this information. These results are in line with the report of Khanal et al. [8], who observed a higher predictive ability of models that included both genetic and microbiome effects for most of the traits.

In addition, lower accuracy of microbiome predictions of phenotypes were obtained with the CG designs than with the random designs. This could be due to the high impact of the contemporary environment on the microbiome composition of animals. Vigors et al. [29] reported that microbiome composition can vary between farms because of differences in management, season of the year, and sanitary status, and of the influence of the dam’s diet. A preliminary non-metric multidimensional scaling analysis revealed that CG was the main driving factor of the microbiota composition in our dataset. Given that, in our study, the samples were collected on an experimental farm under a standardized management protocol, microbiota differences between CG should be limited compared to differences between farms. Therefore, including data of animals sharing the same breeding environment as the predicted animals in the training sets may provide more accurate predictions of phenotypes when using the microbiome information. In conclusion, homogenous breeding conditions between training and predicted populations, at least at the farm level, are suggested to obtain reliable microbiome predictions. This concept can also justify combining both the microbiota and genetic components in prediction models, since similar values of $\overline{\mathrm{r }\left({(\widehat{\mathbf{a}+\mathbf{m}})}_{\mathbf{p}},{(\widehat{\mathbf{a}+\mathbf{m}})}_{\mathbf{w}}\right)}$ were obtained between CG and random designs: the genetic component could then stabilize the predictions across varying environments.

### Microbiome-wide association studies

As expected from previous studies [23, 25], the results of the two approaches used here to detect the association between OTU and the phenotypic traits were highly consistent but detected slightly different numbers of associated OTU. Both methods detected four OTU associated with the traits, and the single-OTU regression pointed out four suggestive OTU whereas the back-solving method identified two. This slight difference could be due to the properties of the BLUP method, which tends to shrink the effect solutions towards the mean of the population, shrinkage that can be passed on to the estimates of the OTU effects after the back-solving. However, single-effect regression and BLUP-based methods have been shown to have an equivalent power for the association studies [30], and the same conclusion seems plausible for MWAS. In addition, the detection power in GWAS is in great part due to the extent of the linkage disequilibrium between SNPs. The compositional nature of the OTU data, i.e. the fact that OTU are constrained by an arbitrary total number of sequences [31], can be assumed to have similar effects on MWAS, as it creates a correlation structure between OTU abundancies. This correlation structure would affect both approaches, and further studies are needed to understand and explain how it would impact MWAS results and to evaluate if data that are pre-transformed to break the compositional nature of the OTU abundances would result in different outcomes.

Even with low values of microbiome variances estimated from Model (4) for DFI, ADG and BFT, the MWAS results were equivalent between the single-OTU regression and the back-solving approaches, which can be considered as an indirect confirmation of the estimated microbiome variances. Indeed, if variances deviated strongly from the actual values, biased MWAS results could be expected from the back-solving approach.

The tests indicated that the abundance of some of the OTU may be associated with the variability of phenotypic traits. We have previously shown that some microbiota genera differ between lines [12]. The aim of including the genetic covariance matrix in the model to run MWAS was to control the risk that the association of abundance of the significant OTU with phenotypes would be due to the divergent selection, since we could not conduct separate analyses for each pig line because their power would have been too limited.

In a previous study at the genera level, using the same microbiome dataset but filtered for the most abundant genera, we showed significant genetic correlations of abundance of genera from the *Lachnospiraceae*, *Ruminococcaceae*, *Prevotellaceae* and *Streptococcaceae* families with RFI, DFI, and BFT [12]. Our findings that abundances of OTU from these families are significantly associated with phenotypic traits are consistent with these previous results. However, the negative estimate of the regression coefficient for the OTU pertaining to the *Streptococcus* genus in our present MWAS is opposite to the positive genetic correlation that was estimated between RFI and the *Streptococcus* genus in our earlier study. This suggests potential antagonistic relationships between microbiota and traits at the phenotypic and genetic levels. Weishaar et al. [27] also reported that abundance of OTU from the *Lachnospiraceae* and *Prevotellaceae* families had strong effects on FCR and RFI, but without indication of the direction of these effects. The *Prevotellaceae*, *Lachnospiraceae* and *Ruminococcaceae* families are involved in the digestion of fibrous material and provide short-chain fatty acids to the host [32, 33]. Bacteria from the *Streptococcaceae* family are known to be lactic acid producer bacteria [34] that have an important role in the production of dietary enzymes, such as amylase, lipase, phytase, and protease [35]. Therefore, the OTU that were identified in the MWAS could have meaningful biological links with feed efficiency and other performance traits. We did not find any OTU that was strongly associated with more than one trait and that could be claimed as a “major OTU” affecting production trait. In addition, most of these OTU were rare, with only one, OTU391, appearing to be sufficiently abundant in our conditions to be quantified systematically with a reasonable sequencing depth. If this link between OTU391 and RFI was confirmed in more diverse conditions and at the genetic level, this OTU could be used as a biomarker in selection programs to improve feed efficiency in pigs. As a next step, it will be necessary to evaluate how accounting for the microbiota composition in linear mixed models will improve the prediction accuracies of breeding values.

## Conclusions

We have shown that microbiota information can be used to better predict traits in pigs, especially feed efficiency traits. The sizable ${\mathrm{m}}^{2}$ and the identification of some OTU with abundances that are associated with the phenotype traits indicate that some of the microbiota components are associated with the variability of production traits. In addition, the lower accuracy of microbiome predictions of phenotypes when none of the individuals from the contemporary group of the pigs were included in the training set suggests that connecting breeding conditions between the training and predicted datasets is needed. Altogether, prediction accuracies of phenotypes accounting for microbiome and genetic covariance between animals suggest that phenotypes of animals can be reliably predicted at mid-test. These outcomes need to be confirmed in more diverse datasets with different environmental factors that may strongly modify the microbiota composition, in order to identify the limits of the use of microbiota information for the prediction of phenotypes in pigs.

## Supplementary Information

**Additional file 1: Figure S1.** Average correlations between ${\widehat{\mathbf{m}}}_{\mathbf{p}}$ ($\widehat{\mathbf{m}}$ using a partial dataset) and ${\widehat{\mathbf{m}}}_{\mathbf{w}}$ ($\widehat{\mathbf{m}}$ using the whole dataset) for the CG design, and their SD as error bars**. Figure S2.** Average correlations between ${(\widehat{\mathbf{a}+\mathbf{m}})}_{\mathbf{p}}$ and ${(\widehat{\mathbf{a}+\mathbf{m}})}_{\mathbf{w}}$ for CG designs, and their SD as error bars. **Figure S3.** Results of microbiome wide association analyses using back solving of BLUP solutions between operational taxonomic units and residual feed intake (RFI), feed conversion ratio (FCR), daily feed intake (DFI), average daily gain (ADG) and back fat thickness (BFT).

## Data Availability

The sequences are available at the Bioproject database https://www.ncbi.nlm.nih.gov/bioproject/) with accession number PRJNA701065. Other data are available upon reasonable request to the authors.
