# Supplementary material for: Microbiability and microbiome-wide association analyses of feed efficiency and performance traits in pigs
Source: Genet Sel Evol. 2022 Apr 25;54:29. doi: 10.1186/s12711-022-00717-7 (PMC9036775; doi:10.1186/s12711-022-00717-7)
Supplement: Supplementary file 1 — Additional file 1: Figure S1. Average correlations between ${\widehat{\mathbf{m}}}_{\mathbf{p}}$m^p ($\widehat{\mathbf{m}}$m^ using a partial dataset) and ${\widehat{\mathbf{m}}}_{\mathbf{w}}$m^w ($\widehat{\mathbf{m}}$m^ using the whole dataset) for the CG design, and their SD as error bars. Figure S2. Average correlations between ${(\widehat{\mathbf{a}+\mathbf{m}})}_{\mathbf{p}}$(a+m^)p and ${(\widehat{\mathbf{a}+\mathbf{m}})}_{\mathbf{w}}$(a+m^)w for CG designs, and their SD as error bars. Figure S3. Results of microbiome wide [file 12711_2022_717_MOESM1_ESM.docx]

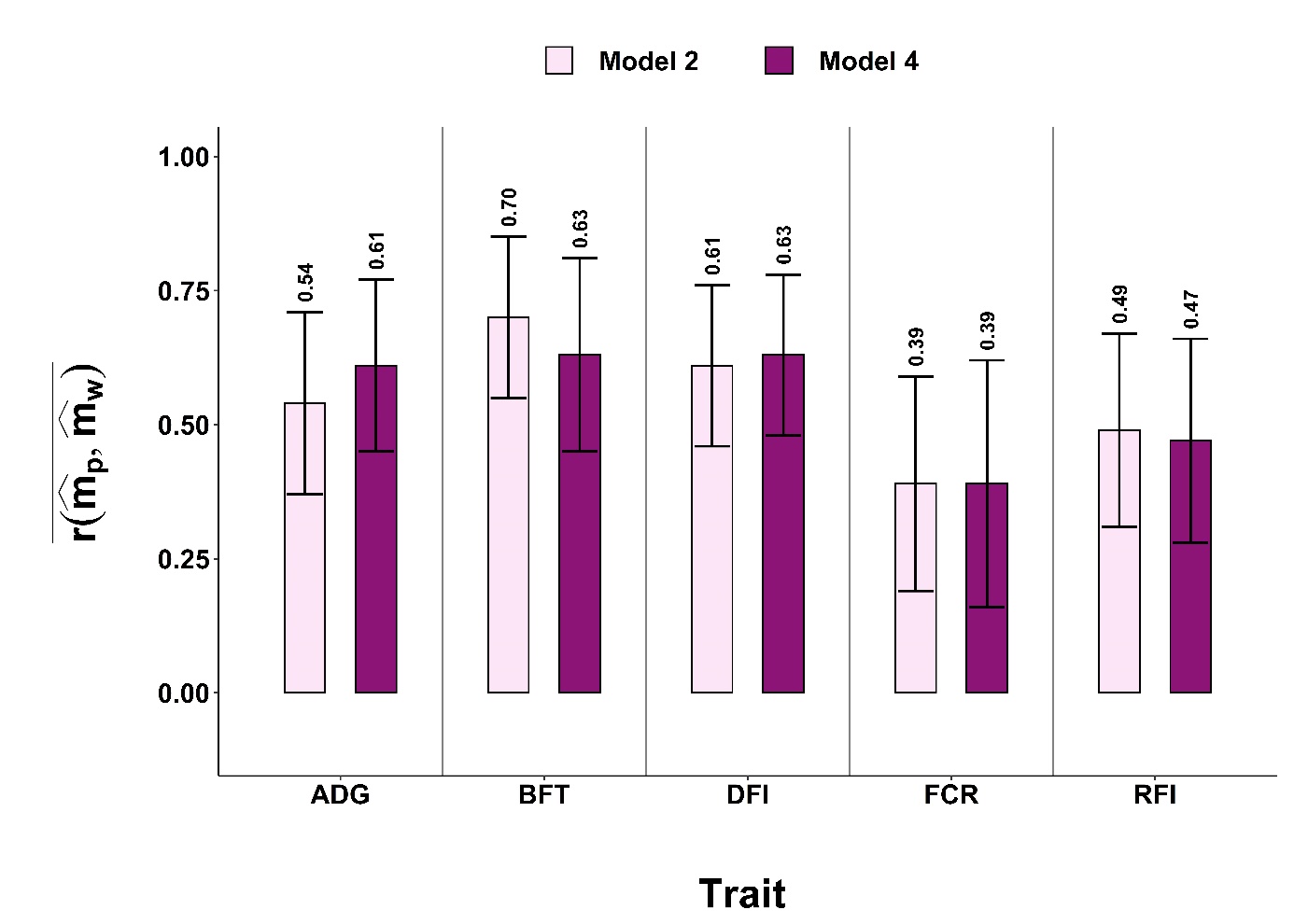


Figure S1. Average correlations between ${\hat{\mathbf{m}}}_{\mathbf{p}}$ ($\hat{\mathbf{m}}$ using a partial dataset) and ${\hat{\mathbf{m}}}_{\mathbf{w}}$ ($\hat{\mathbf{m}}$ using the whole dataset) for the CG design, and their SD as error bars

ADG average daily gain, BFT backfat thickness, DFI daily feed intake, FCR feed conversion ratio, RFI residual feed intake, EMV estimated microbiota values


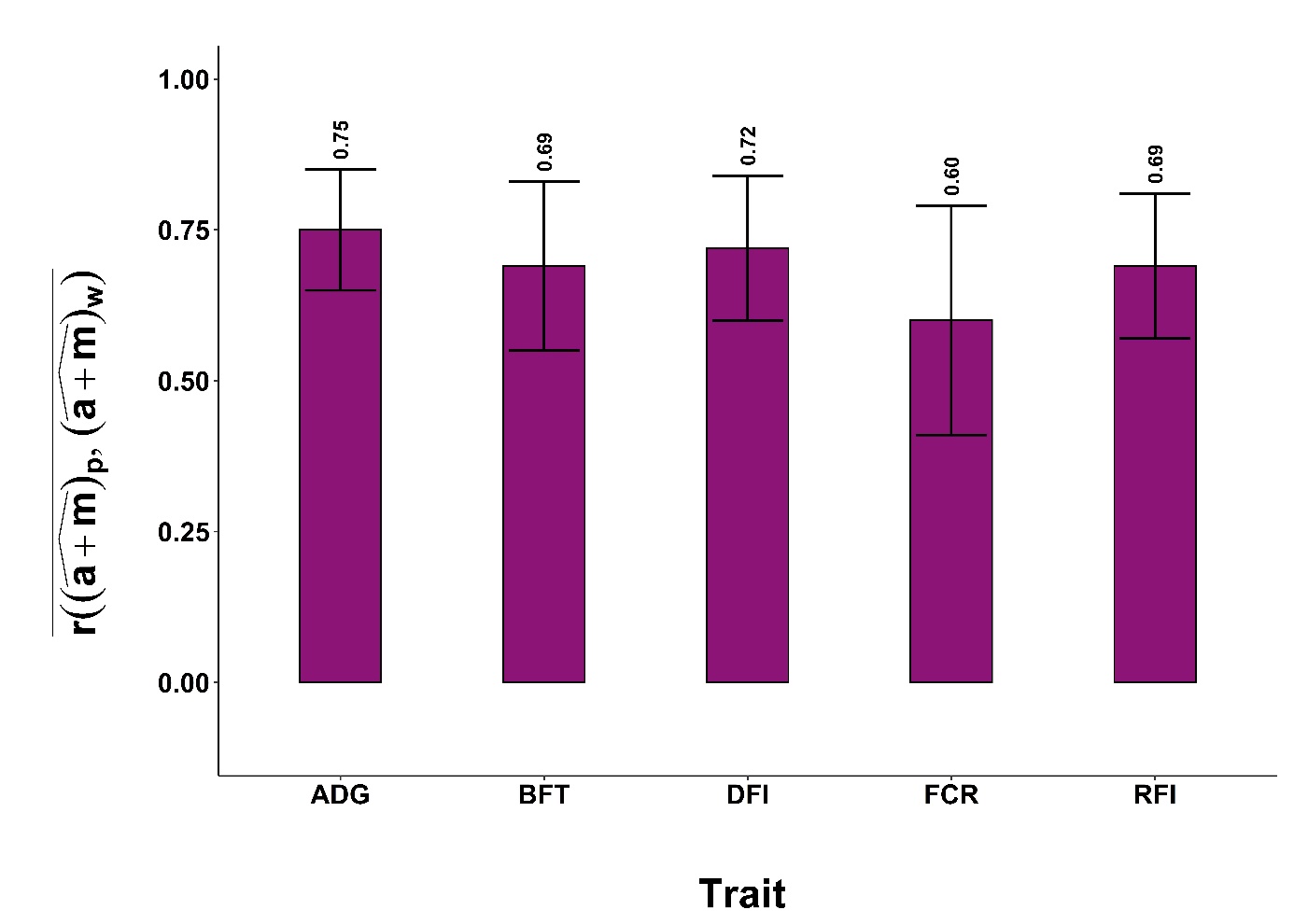


Figure S2. Average correlations between ${\mathbf{(}\hat{\mathbf{a+m}}\mathbf{)}}_{\mathbf{p}}$ and ${\mathbf{(}\hat{\mathbf{a+m}}\mathbf{)}}_{\mathbf{w}}$ for CG designs, and their SD as error bars

ADG average daily gain, BFT backfat thickness, DFI daily feed intake, FCR feed conversion ratio, RFI residual feed intake


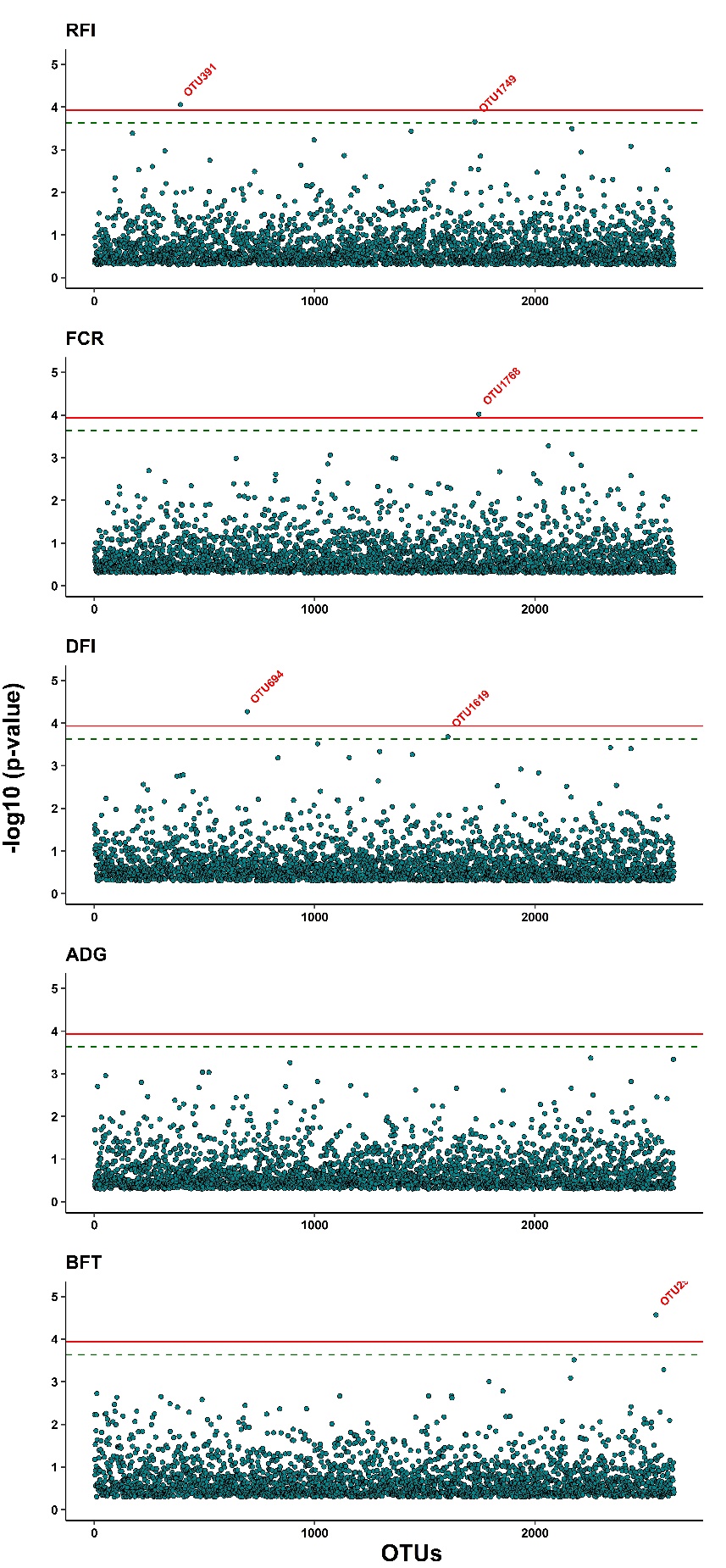


Figure S3. Results of microbiome wide association analyses using back solving of BLUP solutions between operational taxonomic units and residual feed intake (RFI), feed conversion ratio (FCR), daily feed intake (DFI), average daily gain (ADG) and back fat thickness (BFT). In the plots, the solid and dashed lines represent significance and suggestive significance at 5% and 10% family-wise type I error rates, respectively.
